# Association between serum selenium level and type 2 diabetes mellitus: a non-linear dose–response meta-analysis of observational studies

**DOI:** 10.1186/s12937-016-0169-6

**Published:** 2016-05-04

**Authors:** Xin-liang Wang, Tu-bao Yang, Jie Wei, Guang-hua Lei, Chao Zeng

**Affiliations:** 1 Department of Epidemiology and Health Statistics, Xiangya School of Public Health, Central South University, Changsha, 410008 Hunan Province China; 2 Health Management Center, Xiangya Hospital, Central South University, Changsha, 410008 Hunan Province China; 3 Department of Orthopaedics, Xiangya Hospital, Central South University, Changsha, 410008 Hunan Province China

**Keywords:** Selenium, Type 2 diabetes mellitus, Observational studies, Non-linear dose–response meta-analysis

## Abstract

**Background:**

The association between serum selenium levels and type 2 diabetes mellitus (T2DM) is controversial. We performed a systematic review and non-linear dose–response meta-analysis of observational studies to investigate the association in the present study.

**Methods:**

A comprehensive literature search was conducted using MEDLINE and EMBASE databases. A pooled odds ratio (OR) and related 95 % confidence interval (95 % CI) for T2DM between the highest and lowest serum selenium categories, and a non-linear dose–response relationship between selenium and T2DM were estimated.

**Results:**

A total of five studies (of 13,460 participants) were identified as meeting the inclusion criteria. The pooled OR indicated that there was a significantly higher prevalence of T2DM in the highest category of blood selenium compared with the lowest (OR = 1.63, 95 % CI: 1.04–2.56, *P* = 0.033). Moreover, a significant non-linear dose–response relationship was observed between serum selenium levels and T2DM (*P* < 0.001). Serum selenium levels were positively associated with T2DM in populations with relatively low serum selenium levels (<97.5 μg/l) and those with high serum selenium levels (>132.5 μg/l).

**Conclusions:**

The positive association between serum selenium levels and T2DM existed in populations with relatively low levels and high levels of serum selenium, indicating a likely U-shaped non-linear dose–response relationship between serum selenium and T2DM.

**Electronic supplementary material:**

The online version of this article (doi:10.1186/s12937-016-0169-6) contains supplementary material, which is available to authorized users.

## Background

Selenium is one of the most essential trace elements for human health. It functions as a redox center as part of the family of selenium-dependent glutathione peroxidases (GPx), transforming hydrogen peroxide and damaging lipid and phospholipid hydroperoxides into harmless products [1–3]. It is also a basic component of selenoproteins, which are a group of critically important enzymes [1]. In its protective role against oxidative stress, selenium has drawn increasing attention for preventing type 2 diabetes mellitus (T2DM), cardiovascular disease, cancer, and other chronic diseases [4–6]. Low selenium levels are thought to contribute to disease etiology, or may be a consequence of disease that aggravates the condition further [1].

T2DM is the most common form of diabetes, and is caused by a progressive insulin secretory defect against a background of insulin resistance [7]. Its relationship with oxidative stress may reflect the excess levels of reactive oxygen species in hyperglycemia. Oxidative stress has a huge impact on the etiology, pathogenesis, and complications of T2DM [8, 9]. In view of the protective function of selenium against oxidative stress, selenium might be expected to play a protective role against T2DM. However, the relationship between selenium and T2DM is highly complex. The over-expression of GPx in islets may protect pancreatic β cells from oxidative stress, stimulate pancreatic β cell gene expression, and improve islet function [10–12]. Conversely, high selenium concentrations in the human body may interfere with insulin signaling, which is critical to the regulation of glucose levels and the prevention of diabetes [13, 14].

Many researchers have investigated the effects of blood selenium levels on T2DM, but the conclusions are controversial. Some studies report that blood selenium is negatively associated with T2DM [15–23], while others indicate a positive correlation [24–28]. Meanwhile, no significant association is documented by other reports [29–34]. Because of this complicated relationship between selenium and T2DM, we hypothesized that it might not be linear. Additionally, the selenium status and intake vary widely in different parts of the world [1], which might cause the inconsistencies observed in study outcomes.

A previous review article [5] suggested that there might be no standard or specific definition for serum or plasma selenium deficiencies. However, recommendations for dietary selenium intake are available, advising an average of 60 μg per day for men and 53 μg per day for women [35]. Several previous studies indicated that individuals with serum or plasma selenium concentrations of 122 μg/L or higher should not supplement with selenium [36, 37]. Conversely, those with serum or plasma selenium concentrations lower than 122 μg/L could benefit from increased levels (up to 130–150 μg/L), which would reduce their mortality [38]. However, these findings were based on qualitative estimations, and the relationship between serum selenium levels and T2DM remains unclear.

Therefore, the objective of the present research was to comprehensively review the evidence on the association between serum selenium levels and T2DM by conducting a non-linear dose–response meta-analysis of observational studies.

## Methods

### Search strategy

This meta-analysis was conducted following Preferred Reporting Items for Systematic Reviews and Meta-analyses guidelines. MEDLINE and EMBASE databases were searched in September 2015 to identify relevant observational studies that investigated the relationship between blood selenium levels and T2DM. Search key words included “diabetes mellitus”, “T2DM”, “NIDDM”, and “selenium”. The specific search strategies are listed in Additional file 1: Appendix 1.

### Study selection

Two researchers independently reviewed all citations and abstracts generated by the literature search. Qualified studies were subsequently examined for inclusion. Each researcher independently assessed the full texts in a blinded and standardized manner using a customized form to determine whether all inclusion criteria were met. Disagreements were resolved by discussion to reach a consensus.

Studies that met the following criteria were included: 1) observational comparative studies relating to the association between blood selenium levels and T2DM; 2) patients diagnosed with confirmed T2DM; 3) the exposure of interest was serum selenium levels, and at least three quantitative categories of selenium levels were classified; 4) the selenium level for each response category was provided or could be calculated; 5) the study reported numbers of cases and controls for each serum selenium category; and 6) full research articles. Exclusion criteria were: 1) patients with type 1 diabetes mellitus or gestational diabetes; 2) randomized controlled trials investigating the effect of selenium supplementation on T2DM; 3) review articles; 4) in vitro studies and animal experiments; and 5) short reports or meeting abstracts.

### Data extraction and quality assessment

The following background information was extracted from the included studies: first author, publication year, country, study design, setting, study population, sample size, basic characteristics of participants (age, sex, and body mass index), and selenium classification. Data on the number of cases and participants in different selenium categories, and the odds ratios (ORs) of incidence/prevalence of T2DM between the highest and the lowest selenium category were extracted for analysis.

The methodological quality of included studies was evaluated by the Newcastle–Ottawa Scale (NOS) [39], which was developed to assess the quality of non-randomized studies based on three broad perspectives: selection of the study groups; comparability among different groups; and the ascertainment of either the exposure or outcome of interest. The total NOS score ranged from 0 to 9 based on the assessment items. Studies scoring higher than 6 were considered to have a high methodological quality.

### Statistical analysis

The association between blood selenium levels and T2DM was evaluated by the pooled OR and related 95 % confidence interval (95 % CI) for T2DM between the highest and lowest selenium categories. The heterogeneity of effect size across studies was tested by the Q statistic (*P* < 0.05 was considered heterogeneous) or *I* ^2^ statistic (*I* ^2^ > 50 % was considered heterogeneous). The random effects model was used if significant heterogeneity was detected among studies; otherwise, the fixed effects model was applied. Sensitivity analysis was conducted to examine the influence of specific exclusion criteria on the overall effect size. Begg’s tests [40] and funnel plots were performed to assess publication bias.

A two-stage random effects dose–response meta-analysis was conducted to investigate the potential non-linear dose–response relationship between blood selenium levels and T2DM [41]. First, a restricted cubic splines model with four knots at percentiles 5, 35, 65, and 95 % of the distribution was adopted using generalized least squares regression. Then, the restricted maximum likelihood method in a random effects meta-analysis was adopted to calculate the pooled study-specific estimates [42–44]. The median or mean selenium concentration for each category was assigned to each corresponding OR with 95 % CI. When only the range of the selenium concentration was reported, the mid-point of the range was adopted to represent the category. When the category was open-ended, we assumed the open-ended interval length was the same as the adjacent interval [45, 46].

Statistical analyses were performed using STATA version 11.0 (StataCorp LP, College Station, TX). *P* < 0.05 was considered to be statistically significant.

### Statistical analysis

The association between blood selenium level and T2DM were evaluated by a pooled OR and related 95 % confidence interval (95 % CI) for T2DM between the highest and the lowest selenium category. The heterogeneity of effect size across studies was tested by Q statistic (p < 0.05 was considered heterogeneous) or I^2^ statistic (I^2^ > 50 % was considered heterogeneous). If there was a significant heterogeneity among studies, the random-effects model was used; otherwise, the fixed-effects model was applied. A sensitivity analysis was conducted to examine the influence of specific exclusion criteria on the overall effect sizes. Begg’s tests [40] and funnel plot was performed to assess the publication bias.

For the potential non-linear dose–response relationship between blood selenium level and T2DM, a 2-stage, random effects, dose–response meta-analysis was conducted [41]. Firstly, restricted cubic splines model with four knots at percentiles 5, 35, 65 and 95 % of the distribution was adopted by using generalized least squares regression. Then the restricted maximum likelihood method in a random-effects meta-analysis was adopted to calculate the pooled study-specific estimates [42–44]. The median or mean selenium concentration for each category was assigned to each corresponding ORs with 95 % CI. When only range of selenium concentration was reported, the mid-point of the range was adopted for representing the category. When the category was open-ended, we assumed the open-ended interval length to be the same as the adjacent interval [45, 46].

Statistical analyses were performed using STATA version 11.0 (StataCorp LP, College Station, Texas). *P* < 0.05 was considered to be statistically significant.

## Results

### Literature search

**Fig. 1:**
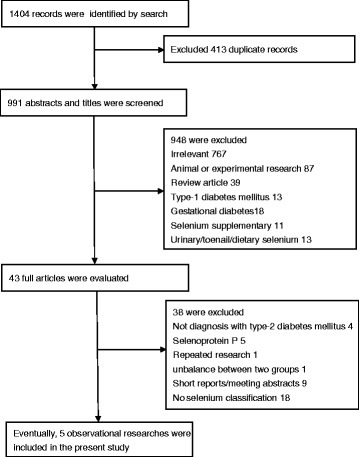
Flow chart of the selection process of the included studies

Figure 1 shows the selection process of the observational studies included in this study. A total of 1404 records were selected following the search strategies. After removing duplicate records, 991 abstracts and titles were initially identified. Of these, 948 records were excluded because they did not meet inclusion criteria, and the remaining 43 full-text studies were evaluated for eligibility. Eventually, a total of five studies were included in this research.

### Study characteristics

The characteristics of the included studies and subjects are shown in Table 1. A total of 13,460 participants were included within the five studies [25, 27, 28, 32, 47]. The studies were published from 1998 to 2015; two were conducted in the United States [25, 27], two in Sweden [32, 47], and one in Italy [28]. Three studies were longitudinal studies [28, 32, 47], but two of these were considered to be cross-sectional because the data were extracted from baseline [28, 47]. The remaining two studies were also cross-sectional [25, 27]. The community population was targeted in all included studies, and the sample size varied from 445 to 8876. The methodological quality, based on NOS, is reported in Table 2. All studies were evaluated with a high methodological quality: three studies scored 9 and two studies scored 7.

**Table 1:** Characteristics of studies included in the meta-analysis

First author, year and country	Study design	Setting, study population	Sample size (n)	Age, mean	Male (%)	BMI (kg/m^2^), mean	Selenium classification (range)	Definition of T2DM
Alehagen 2015, Sweden	Longitudinal study (data were extracted from baseline)	Community, rural municipality of inhabitants in the south-east of Sweden.	668	77.7	47.8	NR	Quartiles: <57.2, 57.2–67.1, 67.1–76.1, >76.1 μg/l	NR
Bleys 2007, USA	Cross-sectional study	Community, NHANES III participants	8876	45.2	49.1	26.9	Quintiles: <111.62, 111.62–120.17, 120.18–128.25, 128.26–137.65, >137.65 μg/l	Defined as a fasting plasma glucose ≥ 126 mg/dl, a self-report of a physician diagnosis of diabetes, or current use of insulin or oral hypoglycemic medication
Laclaustra 2009, USA	Cross-sectional study	Community, civilian non-institutionalized U.S. population from NHANES 2003–2004	890	54.4	47.0	28.8	Quartiles: <124, 124–133, 134–146, >146 μg/l	Defined as the presence of either a self-report of current use of hypoglycemic agents or insulin or fasting plasma glucose ≥ 126 mg/dL.
Stranges 2011, Italy	Longitudinal study (data were extracted from baseline)	Community, male workforce of the Olivetti factories in Pozzuoli (Naples) and Marcianise (Caserta), Southern Italy.	445	50.9	100	26.8	Tertiles: <69.7, 69.7–82.6, > 82.6 μg/l	Defined as a fasting blood glucose level ≥ 126 mg/dL or anti-diabetic therapy.
Gao 2014, Sweden	Longitudinal Study	Community, all 50-year-old men living in Uppsala County, Sweden.	1925	49.7	100	25.0	Tertiles: 61.4, 75.1, 91.1 μg/l^a^	Defined by elevated fasting glucose levels and/or use of anti-diabetic medicine. Elevated glucose levels were assessed as fasting blood glucose ≥6.1 mmol/l at the age 60 years’ examination (equals ≥7.0 mmol/l of plasma glucose) and fasting plasma glucose ≥ 7.0 mmol/l at age 70 years and age 77 years

*Abbreviations*: *NHANES* National Health and Nutrition Examination Survey, *NR* not report

^a^: number represents median level of each category

**Table 2:** Methodological quality assessment according to Newcastle-Ottawa Scale (NOS)

Study	Selection				Comparability	Exposure/outcome			Total score
	Adequate definition of case/exposed cohort	Representativeness of the cases/ exposed cohort	Selection of Controls	Definition of Controls	Comparability of cases and controls (worth 2 points)	Ascertainment of exposure/outcome	Same method of ascertainment for cases and controls/long enough for outcomes to occur	Non-Response Rate	
Alehagen 2015	1	1	1	1	0	1	1	1	7
Bleys 2007	1	1	1	1	2	1	1	1	9
Gao 2014	1	1	1	1	2	1	1	1	9
Laclaustra 2009	1	1	1	1	2	1	1	1	9
Stranges 2011	1	1	1	1	0	1	1	1	7

### OR for T2DM between different serum selenium categories

**Fig. 2:**
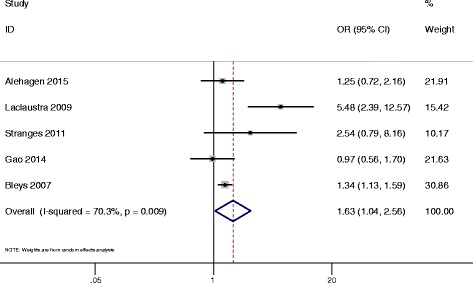
Forest plot of OR for T2DM between the highest and lowest serum selenium category

The pooled data analysis indicated that there was a significantly higher prevalence of T2DM in the highest category of blood selenium compared with the lowest (OR = 1.63, 95 % CI: 1.04–2.56, *P* = 0.033, Fig. 2). Unfortunately, significant heterogeneity was observed among different studies (*P* = 0.009, *I* ^2^ = 70.3 %). No evidence of publication bias was observed according to the Begg rank-correlation test among the included studies (*P* = 0.221). The Begg’s funnel plot is shown in Additional file 1: Appendix 2.

**Fig. 3:**
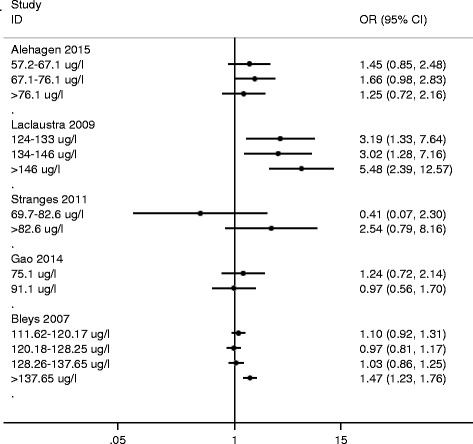
Forest plot of OR for T2DM between different serum selenium categories and the lowest category (reference) in individual study

Because of the substantial degree of heterogeneity among studies, a qualitative analysis was adopted. As shown in Fig. 3, a significant association was observed between high serum selenium concentrations and increased prevalence of T2DM in studies including participants with relatively high selenium concentrations [25, 27]. Studies conducted in participants with relatively lower selenium concentrations did not show a significant association between serum selenium concentration and T2DM [28, 32, 47]. We also performed a sensitivity analysis by excluding one study [27] that was an outlier; the outcome suggested a similar positive association between T2DM and serum selenium (OR = 1.32, 95 % CI: 1.12–1.54, *P* = 0.001) but with a significantly decreased level of heterogeneity (*P* = 0.495, *I* ^2^ = 0 %).

### Non-linear dose–response relationship between blood selenium and T2DM

**Fig. 4:**
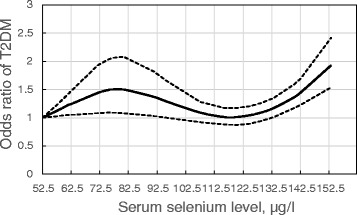
Nonlinear dose–response relationship between selenium and T2DM. The middle line represents the odds ratio of T2DM compared with reference (53.5 ug/l), and the upper and lower lines represent 95 % CI of the odds ratio

A significant non-linear dose–response relationship between blood selenium levels and T2DM was found (*P* < 0.001 for nonlinearity; Fig. 4). The reference selenium level was 52.5 μg/l. When serum selenium concentrations exceeded 132.5 μg/l, they were strongly positively associated with increased prevalence of T2DM. There was also a moderately positive association between serum selenium and T2DM when individuals had relatively low levels of serum selenium (<97.5 μg/l). However, the positive association began to disappear with a flattening of the curve at the middle level (97.5–132.5 μg/l) of serum selenium.

## Discussion

To the best of our knowledge, this is the first systematic review and dose–response meta-analysis examining the association between serum selenium levels and T2DM based on previous observational studies. We found a significantly higher prevalence of T2DM in the highest category of blood selenium compared with the lowest, and serum selenium levels were positively associated with T2DM in populations with relatively low (<97.5 μg/l) or high serum selenium levels (>132.50 μg/l).

Previous evidence about the relationship between selenium and T2DM is conflicting. Some studies claim that patients with T2DM have significantly lower selenium concentrations compared with healthy subjects [15–23]. Antioxidants in non-diabetic individuals can mitigate free radical-mediated oxidative stress, but in the chronic hyperglycemia state, increased free radical production may lead to the overconsumption of chain-breaking antioxidants, elevated lipid peroxidation, and enhanced oxidative stress [22]. Selenium is believed to be one of the most important antioxidant nutrients in the human body, and selenoproteins have a protective effect against oxidative stress and inflammation [5, 8]. However, the lower selenium status measured in T2DM populations of some case–control studies might be caused by the disease and its association with inflammation. Other previous studies have suggested that the inflammatory response decreases the expression of selenoprotein P and reduces plasma selenium concentrations [48, 49].

Conversely, some studies report that patients with T2DM have higher selenium concentrations compared with healthy subjects, and that blood selenium levels positively correlate with T2DM [24–28]. The over-expression of GPx1 in transgenic mice was found to lead to the development of insulin resistance, hyperglycemia, hyperinsulinemia, and obesity. After knocking out GPx1, both insulin-induced glucose uptake and insulin sensitivity were improved [50–52]. This was explained by GPx1 removing hydrogen peroxide, a second messenger in the insulin signaling cascade, and insulin binding to its receptor [14]. The same correlation was also observed between increased erythrocyte GPx1 activity and mild insulin resistance in pregnant women [53]. Another selenoprotein, SeP, was also shown to be related to insulin resistance by aggravating insulin resistance through impairing insulin signaling [54]. Studies have revealed a significantly positive association between SeP and fasting plasma glucose in Japanese and Korean populations [54, 55]. Moreover, in an earlier study, we found a significant positive correlation between dietary selenium intake and the prevalence of diabetes [56].

Previous review articles have suggested that the association between selenium and T2DM is U-shaped, such that selenoprotein levels both below and above the physiological range are possible risk factors for T2DM [5, 8, 12]. This estimation is consistent with our non-linear dose–response analysis. We observed a moderately positive association between selenium and T2DM in individuals with relatively low levels of serum selenium (<100 μg/l), a non-significant association between selenium and T2DM in individuals with middle levels of serum selenium (100–130 μg/l), and a strongly positive association in individuals with relatively high levels of serum selenium (>130 μg/l). Stranges et al. [37] concluded that selenium supplementation does not prevent T2DM, but may instead increase the disease risk in populations with a median serum selenium level of 136 μg/l. Another study suggested that both high and low expression levels of GPx1 and other related selenoproteins are harmful because of their effects on insulin resistance and hyperglycemia [57].

The present study has several strengths. It is the first meta-analysis to perform a systematic and quantitative analysis of the association between serum/plasma selenium levels and T2DM. Several review articles [5, 8, 12] have attempted to examine this relationship, but none performed a quantitative analysis. Second, this study showed a specific non-linear dose–response relationship between serum selenium levels and T2DM. However, the limitations of the present study should also be acknowledged. The number of studies included in the analysis was very limited. Second, the shortage of prospective cohort studies could explain the causal relationship between selenium and T2DM. Therefore, future prospective cohort studies are needed to further explore this relationship. Finally, because glucose levels vary greatly in patients with T2DM, direct correlations of selenium levels with glucose levels would provide us with relevant information. Unfortunately, only two studies [27, 32] have reported these data. Additionally, the search strategy and included studies required for these analyses would differ from those of the present meta-analysis. Thus, this would best be examined as part of a future study.

## Conclusion

In conclusion, we detected a positive association between serum selenium levels and T2DM in populations with relatively low and high serum levels of selenium, revealing a U-shaped non-linear dose–response relationship between serum selenium and T2DM.

## Additional file

Additional file 1: Appendix 1. Search strategies. **Appendix 2.** Beggs’funnel plot. (DOCX 18 kb)
