# Supplementary material for: Association between serum selenium level and type 2 diabetes mellitus: a non-linear dose–response meta-analysis of observational studies
Source: Nutr J. 2016 May 4;15:48. doi: 10.1186/s12937-016-0169-6 (PMC4855440; doi:10.1186/s12937-016-0169-6)
Supplement: Additional file 1: Appendix 1. — Search strategies. Appendix 2. Beggs’funnel plot. (DOCX 18 kb) [file 12937_2016_169_MOESM1_ESM.docx]

**Appendix 1 Search strategies**

***Ovid/Medline***

1. exp Diabetes Mellitus/
2. diabet$.tw,ot.
3. (IDDM or NIDDM or MODY or T1DM or T2DM or T1D or T2D).tw,ot.
4. ((non insulin$ depend$) or (noninsulin$ depend$) or (non insulin?depend$) or noninsulin?depend$).tw,ot.
5. (insulin$ depend$ or insulin?depend$).tw,ot.
6. exp Diabetes Insipidus/
7. diabet$ insipidus.tw,ot.
8. OR/1-7
9. exp selenium/
10. exp selenomethionine/
11. (selenium or selenomethionin* or sodium selenit*).tw,ot.
12. selen*.mp.
13. OR/9-12
14. (retrospective$ or prospective$).ab,ti,tw.
15. ((cross section$) or longitudinal).ab,ti,tw.
16. (((case control) or (case-control)) or cohort).ab,ti,tw.
17. (compare$ or relation$ or associate$ or observ$ or group$).ab,ti,tw.
18. OR/14-17
19. 8 AND 13 AND 18

***Ovid/Embase***

1. exp Diabetes Mellitus/
2. diabet$.tw,ot.
3. (IDDM or NIDDM or MODY or T1DM or T2DM or T1D or T2D).tw,ot.
4. ((non insulin$ depend$) or (noninsulin$ depend$) or (non insulin?depend$) or noninsulin?depend$).tw,ot.
5. (insulin$ depend$ or insulin?depend$).tw,ot.
6. exp Diabetes Insipidus/
7. diabet$ insipidus.tw,ot.
8. OR/1-7
9. exp selenium/
10. exp selenomethionine/
11. (selenium or selenomethionin* or sodium selenit*).tw,ot.
12. selen*.mp.
13. OR/9-12
14. (retrospective$ or prospective$).ab,ti,tw.
15. ((cross section$) or longitudinal).ab,ti,tw.
16. (((case control) or (case-control)) or cohort).ab,ti,tw.
17. (compare$ or relation$ or associate$ or observ$ or group$).ab,ti,tw.
18. OR/14-17
19. 8 AND 13 AND 18

Appendix 2
